# Genetic screening for macular dystrophies in patients clinically diagnosed with dry age‐related macular degeneration

**DOI:** 10.1111/cge.13447

**Published:** 2018-10-15

**Authors:** Eveline Kersten, Maartje J. Geerlings, Marc Pauper, Jordi Corominas, Bjorn Bakker, Lebriz Altay, Sascha Fauser, Eiko K. de Jong, Carel B. Hoyng, Anneke I. den Hollander

**Affiliations:** ^1^ Department of Ophthalmology Donders Institute for Brain, Cognition and Behaviour, Radboud University Medical Center Nijmegen the Netherlands; ^2^ Department of Human Genetics Donders Institute for Brain, Cognition and Behaviour, Radboud University Medical Center Nijmegen the Netherlands; ^3^ Department of Ophthalmology University Hospital of Cologne Cologne Germany; ^4^ Global Head of Ophthalmology F. Hoffmann—La Roche AG Basel Switzerland

**Keywords:** age‐related macular degeneration, AMD, CACD, central areolar choroidal dystrophy, genetic screening, macular dystrophies, *PRPH2*, WES, whole‐exome sequencing

## Abstract

It can be clinically challenging to distinguish dry age‐related macular degeneration (AMD) from AMD‐mimicking dystrophies, and sometimes misdiagnosis occurs. With upcoming therapies for dry AMD it is important to exclude patients with a different retinal disease from clinical trials. In this study we evaluated the occurrence of AMD‐mimicking dystrophies in an AMD cohort. Whole‐exome sequencing (WES) was performed in 218 patients with intermediate AMD or geographic atrophy secondary to AMD and 133 control individuals. WES data was analyzed for rare variants in 19 genes associated with autosomal dominant and recessive macular dystrophies mimicking AMD. In three (1.4%) of 218 cases we identified a pathogenic heterozygous variant (*PRPH2* c.424C > T; p.R142W) causal for autosomal dominant central areolar choroidal dystrophy (CACD). Phenotypically, these patients all presented with geographic atrophy. In 12 (5.5%) of 218 cases we identified a heterozygous variant of unknown clinical significance, but predicted to be highly deleterious, in genes previously associated with autosomal dominant macular dystrophies. The distinction between AMD and AMD‐mimicking dystrophies, such as CACD, can be challenging based on fundus examination alone. Genetic screening for genes associated with macular dystrophies, especially *PRPH2*, can be beneficial to help identify AMD‐mimicking dystrophies.

## 1 INTRODUCTION

Age‐related macular degeneration (AMD) is a common progressive retinal disorder affecting the elderly.^1^ The early stages of AMD are characterized by drusen accumulation in the macula, and as disease progresses two types of advanced AMD can be distinguished: geographic atrophy (GA) and choroidal neovascularization.^2^ Currently, no curative treatment exists for the early and atrophic stages of AMD, which affect the majority of patients (80%‐90%). However, therapies targeting AMD disease pathways are currently being evaluated in clinical trials.^3^, ^4^

In order for clinical trials to be successful, it is crucial to select patients that will most probably benefit from the treatment. However, sometimes it is clinically challenging to distinguish AMD from inherited macular dystrophies.^5^, ^6^, ^7^ Especially when a patient presents at older age and GA has already developed, it can be challenging to distinguish AMD from GA secondary to other macular diseases and potentially patients might be misdiagnosed. Before inclusion of patients in clinical trials for dry AMD, it may therefore be useful to perform genetic testing to exclude AMD‐mimicking dystrophies. In this study, we evaluated the occurrence of rare genetic variants associated with autosomal dominant or autosomal recessive AMD‐mimicking dystrophies in 218 cases diagnosed with dry AMD.

## 2 METHODS

### 2.1 Study population

For this study, we selected patients with intermediate AMD (n = 126) or advanced atrophic AMD (n = 92) from the European Genetic Database (EUGENDA). For 33 cases, one of more family members was included. In total, 62 family members were included, of which 40 were diagnosed with AMD, and 22 did not have signs of AMD. Additionally, 133 control individuals aged 65 years and older without signs of AMD were included in this study. Color fundus photographs of both eyes, and if available spectral domain optical coherence tomograms and fluorescein angiograms, were evaluated by two independent reading center graders according to the Cologne Image Reading Center and Laboratory classification protocol.^8^ All individuals provided written informed consent for enrollment in EUGENDA. This research was approved by the local ethical committees at the Radboud university medical center and the University Hospital of Cologne and the study adhered to the tenets of the Declaration of Helsinki.

### 2.2 Whole‐exome sequencing

Whole‐exome sequencing (WES) was performed as previously described.^9^ WES data were analyzed for rare variants in 19 genes associated previously with autosomal dominant and recessive macular dystrophies mimicking AMD as described by Saksens et al^5^ and RetNet, the Retinal Information Network (Supporting Information Table S1). Filtering of the data was performed to select protein‐altering, nonsense, frameshift or splice‐site variants with a minor allele frequency (MAF) ≤1% in European and Dutch population reference panels.^10^, ^11^ Additional filter criteria included coverage depth of ≥20 reads, ≥10 variant reads and ≥20% variation of reads. A variation of reads between 20% and 80% was defined as heterozygous, and all variants with a variation of reads ≥90% were named homozygous. Individual variants that were seen on less than 25 variant reads were confirmed by Sanger sequencing. Literature and public archives (ClinVar^12^ and LOVD^13^) were consulted to determine if a variant is described to be pathogenic or is of unknown clinical significance (including variants with conflicting interpretations of pathogenicity). We explored the deleteriousness of non‐synonymous missense variants of unknown clinical significance using scaled combined annotation dependent depletion (CADD phred) prediction scores.^14^

## 3 RESULTS AND DISCUSSION

### 3.1 Variants in genes associated with autosomal dominant macular dystrophies

**Figure 1:**
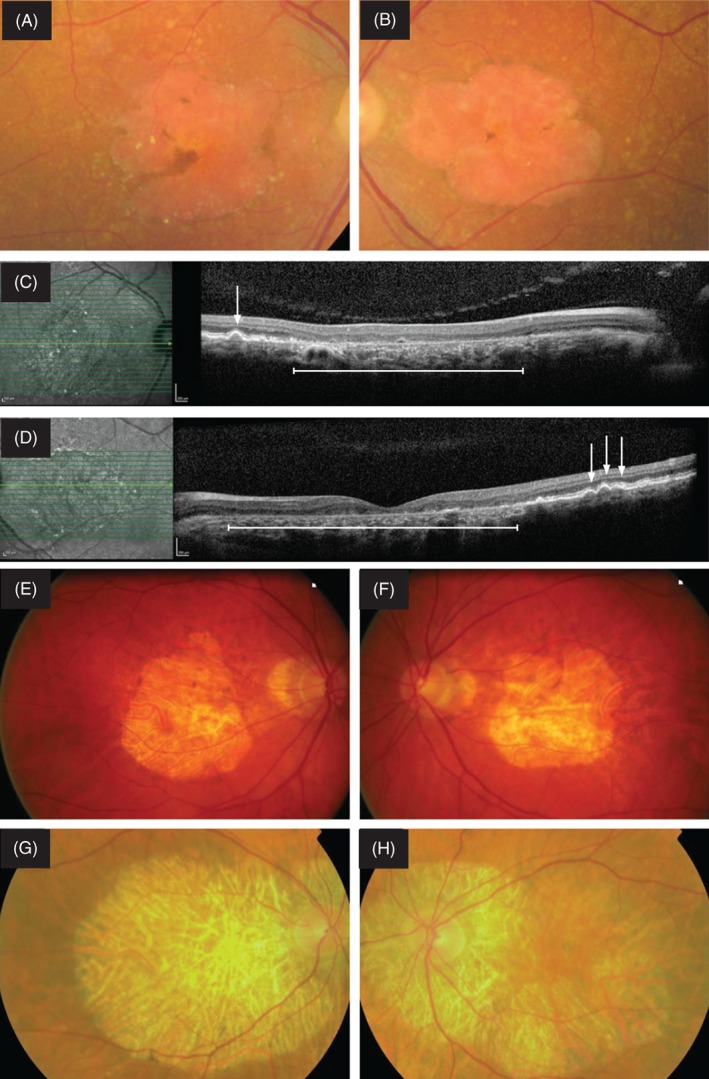
Retinal images of three patients with geographic atrophy secondary to autosomal dominant central areolar choroidal dystrophy (CACD) caused by a heterozygous variant in *PRPH2* (c.424C > T, p.Arg142Trp). Patient 1 (A‐D). Color fundus photographs of right (A) and left (B) eye of a 67‐year‐old female with geographic atrophy and foveal sparing surrounded by drusen secondary to CACD. On optical coherence tomography images of both eyes (C + D) drusen are visible near the edges of the central atrophy. Drusen are indicated by arrows, and atrophy is indicated by a continuous line with dashes just below the atrophic area. Patient 2 (E‐F). A 64‐year‐old male with central atrophy in both eyes secondary to CACD. Patient 3 (G, H). A 76‐year‐old male with extensive geographic atrophy and peripapillary atrophy in both eyes [Colour figure can be viewed at http://wileyonlinelibrary.com]

We identified a heterozygous variant in the *PRPH2* gene (c.424C > T, p.Arg142Trp) in three (1.4%) of 218 patients. All three cases presented with GA which could be secondary to AMD (Table 1, Figure 1), although the area of atrophy in two patients is somewhat larger than would be expected in a typical AMD patient. This variant causes a central cone dystrophy phenotype associated with autosomal dominant central areolar choroidal dystrophy (CACD), and represents a founder mutation in the southeast of the Netherlands.^15^, ^16^ CACD and atrophic AMD have strong phenotypic similarities and their age of onset overlaps.^6^, ^15^, ^16^, ^17^ CACD can be misdiagnosed with AMD based on ophthalmological examination alone, especially in families with incomplete penetrance, which may mask the autosomal dominant inheritance of CACD. Additional imaging, such as spectral‐domain optical coherence tomography and fundus autofluorescence imaging, or genetic analyses, could help distinguish these two diseases.^6^

**Table 1:** Previously described pathogenic variants and variants of unknown clinical significance^a^ in autosomal dominant macular dystrophy genes identified in cases diagnosed with dry AMD

	ExAC MAF (%)	Cases n (MAF %)	CADD score	Disease association	Gender	Age	Phenotypic characteristics on retinal imaging
Known pathogenic variants associated with autosomal dominant macular dystrophy (Figure 1 )							
*PRPH2*							
c.424C > T; p.R142W	0.001	3 (0.69%)	28.6	Central areolar choroidal dystrophy	F M M	67 64 76	GA with foveal sparing surrounded by drusen Central GA Extensive central GA and peripapillary atrophy
Variants of unknown clinical significance in autosomal dominant macular dystrophy (Figure S1)							
***BEST 1***				Adult‐onset foveomacular vitelliform dystrophy Best vitelliform macular dystrophy			
c.1193C > T; p.S398F	0.08	1 (0.23%)	27.8		F	66	Central GA surrounded by small hard drusen extending to the periphery
***ELOVL4***				Stargardt‐like macular dystrophy Autosomal dominant macular dystrophy			
c.145A > G; p.T49A	‐	1 (0.23%)	24.3		F	59	Large soft drusen throughout the macula
***FSCN2***				Autosomal dominant macular degeneration Autosomal dominant retinitis pigmentosa			
c.1057G > A; p.V353 M	0.04	1 (0.23%)	27.9		F	95	Reticular pseudodrusen and some soft drusen
***IMPG1***				Autosomal dominant benign concentric annular macular dystrophy Autosomal dominant vitelliform macular dystrophies			
c.1982G > A; p.R661H	‐	1 (0.23%)	23.5		M	83	Multifocal GA and some intermediate drusen
c.1945C > T; p.L649F	0.40	1 (0.23%)	27.4		F	75	Few intermediate to large soft macular drusen
c.1738C > T; p.R580C	0.02	1 (0.23%)	34		F	70	Intermediate to large soft macular drusen
c.336TC > C; p.I112IX	‐	1 (0.23%)	‐		F	87	Intermediate to large soft macular drusen
***OTX2***				Autosomal dominant pattern dystrophy			
c.844 T > A; p.C282S	0.003	1 (0.23%)	24.3		F	84	Extensive large soft drusen and calcified drusen throughout the macula and reticular pseudodrusen around the retinal arcades
***PRDM13***				North Carolina macular dystrophy			
c.113C > T; p.S38 L	0.07	1 (0.23%)	28.8		M	74	Numerous small hard (cuticular) drusen throughout the macula extending beyond the vascular arches
***PROM1***				Autosomal dominant bull's‐eye macular dystrophy Autosomal dominant stargardt‐like dystrophy			
c.1345G > A; p.V449 M	0.20	1 (0.23%)	20.4		F	79	Central GA surrounded by intermediate to large drusen and some peripheral drusen
c.155 T > C; p.I52T	0.003	1 (0.23%)	23.2		F	81	Drusen deposition throughout the macula
***RP1L1***				Autosomal dominant occult macular dystrophy			
c.553G > T; p.A185S	‐	1 (0.23%)	26.2		F	70	Multifocal GA with foveal sparing surrounded by large soft drusen

Abbreviations: AMD, age‐related macular degeneration; CADD score, combined annotation dependent depletion score; ExAC, Exome Aggregation Consortium; F, female; GA, geographic atrophy; M, male; MAF, minor allele frequency.

a This table includes variants of unknown clinical significance leading to a premature nonsense codon, frameshift, affecting the splice donor or acceptor sites (−1, −2, +1, +2), and non‐synonymous missense variants predicted to be the 1% most deleterious variants in the human genome (CADD score ≥ 20).

In additional, 28 rare variants of unknown clinical significance were identified in other genes associated with autosomal dominant macular dystrophies, while they were not identified in 133 control individuals (Supporting Information Table S2). Because of their uncertain significance further evaluation included only those variants leading to a premature nonsense codon or a frameshift, affecting the invariable splice donor or acceptor sites, and non‐synonymous missense variants predicted to be the 1% most deleterious variants in the human genome (CADD score ≥ 20). We identified 16 variants of unknown clinical significance predicted to be highly deleterious. In four cases, the variants of unknown clinical significance (*CTNNA1* c.536C > T; p.A179V, *FSCN2* c.1025G > A; p.R342Q, *OTX2* c.425C > G; p.P142R, *PROM1* c.2450A > G; p.K817R) did not segregate with the disease in available family members, and were therefore not considered to be pathogenic. The 12 remaining cases carried a variant of unknown clinical significance in the *BEST1*, *ELOVL4*, *FSCN2*, *IMPG1*, *OTX2*, *PRDM13*, *PROM1* or *RP1L1* gene (Table 1). All 12 cases had typical characteristics of intermediate AMD or GA with drusen (Table 1, Supporting Information Figure S1). We cannot rule out the possibility that these variants might be disease‐causing. Therefore, one might consider to exclude patients carrying variants in genes associated with autosomal dominant macular dystrophies from clinical trials, in particular if the disease phenotype matches previously reported disease characteristics of these dystrophies.

### 3.2 Variants in genes associated with autosomal recessive retinal dystrophies

None of our cases carried homozygous or two heterozygous deleterious variants in genes associated with autosomal recessive macular dystrophies. However, 13 (6.0%) out of 218 cases carried a single heterozygous variant, previously described to be pathogenic in the literature or in public archives, in the *ABCA4*, *ABCC6*, *MFSD8* or *PROM1* gene (Table 2, Supporting Information Figure S2). Eight variants showed comparable MAFs with population reference panels, while seven variants were not detected in the 133 control individuals.

**Table 2:** Variants in autosomal recessive macular dystrophy genes previously described as pathogenic

	ExAC MAF (%)	GoNL MAF (%)	Cases Total n = 218 n (MAF %)	Controls Total n = 133 n (MAF%)	Disease association	Gender	Age	Phenotypic characteristics on retinal imaging
*ABCA4*								
c.6089G > A; p.R2030Q	0.06%	0.10%	1 (0.23%)	‐	Stargardt disease	F	35	Extensive drusen deposition, mainly located temporal to the fovea and pigmentary alterations
c.3113C > T; p.A1038V	0.20%	0.30%	1 (0.23%)	‐	Stargardt disease	M	85	Central GA, no evident drusen
c.2947A > G; p.T983A	‐	‐	1 (0.23%)	‐	Stargardt disease	F	72	Small to intermediate hard drusen and pigmentary alterations
c.2588G > C; p.G863A	0.81%	0.80%	4 (0.92%)	1 (0.38%)	Stargardt disease	M F M F	75 78 66 73	Multifocal GA with pigmentary alterations and drusen Extensive drusen deposition (some calcified), mainly located temporal to the fovea and central GA Pigmentary alteration with small to intermediate drusen Confluent soft drusen and minimal central GA
c.2546 T > C; p.V849A	0.01%	‐	1 (0.23%)	‐	Stargardt disease	F	74	Intermediate to large soft drusen throughout the macula
***ABCC6***								
c.2787 + 1G > T; p.?	0.02%	‐	2 (0.46%)	‐	Pseudoxanthoma elasticum	F F	69 66	Central GA surrounded with reticular drusen Minimal pigmentary alterations and small hard drusen
***MFSD8***								
c.1006G > C; p.E336Q	0.33%	0.30%	2 (0.46%)	‐	Non‐syndromic autosomal recessive macular dystrophy	M F	91 71	Multifocal GA with some drusen Central GA without evident drusen
***PROM1***								
c.1355A > TA; p.Y452YX	0.03%	‐	1 (0.23%)	‐	Autosomal recessive cone‐rod dystrophy	F	91	Small to intermediate hard drusen extending beyond the vascular arcades into the periphery

Abbreviations: AMD, age‐related macular degeneration; GA, geographic atrophy; GoNL, Genome of the Netherlands Consortium; ExAC, Exome Aggregation Consortium; MAF, minor allele frequency.

It has been suggested that carriers of a single *ABCA4* variant are at increased risk of developing AMD compared to non‐carriers,^18^, ^19^ although a more recent study described that monoallelic *ABCA4* carriers do not result in retinal changes.^20^ In this study, we identified seven (3.2%) of 218 cases that carried a heterozygous *ABCA4* variant previously reported to be pathogenic, compared to only one (0.8%) of 133 control individuals carrying a heterozygous pathogenic *ABCA4* variant. Larger studies are needed to evaluate the hypothesis that carriers of heterozygous variants associated with autosomal recessive macular dystrophies might be at increased risk for AMD development. The frequency of *ABCA4* variants in our control individuals is, however, lower than expected based on population frequencies, and could also be coincidentally low.

### 3.3 Clinical implications

It is increasingly important to correctly diagnose patients with macular degeneration with respect to inclusion in clinical trials and for future treatment. No curative treatment is currently available for dry AMD, although multiple clinical trials are ongoing.^4^ For clinical trials and future therapies for AMD, it is important to identify those patients that will benefit most probably from the treatment and to exclude AMD‐mimicking dystrophies. Detailed phenotyping is necessary for distinguishing different macular diseases, and multimodal imaging can be useful. Despite modern imaging technologies, however, it can be difficult to clinically differentiate AMD from AMD‐mimicking dystrophies. Genetic screening of genes involved in AMD‐mimicking dystrophies can aid in establishing an accurate diagnosis. Based on the findings of this study, genetic screening of the *PRPH2* gene is recommended because of the significant clinical overlap between CACD and AMD.

## CONFLICT OF INTEREST

Sascha Fauser is an employee of Roche. Anneke den Hollander is a consultant for Ionis Pharmaceuticals.

## Supporting information


**Figure S1.** Clinical imaging of patients with a variant of unknown clinical significance in autosomal dominant macular dystrophy genes identified in cases diagnosed with dry age‐related macular degeneration as listed in Table 1. A, Color fundus photographs (A‐1) and fluorescein angiogram (A‐2) of both eyes of patient with *BEST1* variant (c.1193C > T; p.S398F). B, Color fundus photographs (B‐1) and optical coherence tomography scan (B‐2) of both eyes of patient with *ELOVL4* variant (c.145A > G; p.T49A). C, Color fundus photographs (C‐1) and fluorescein angiogram (C‐2) of both eyes of patient with *FSCN1* variant (c.1057G > A; p.V353M). D, Color fundus photographs (D‐1) and optical coherence tomography scan (D‐2) of both eyes of patient with *IMPG1* variant (c.1982G > A; p.R661H). E, Color fundus photographs (E‐1) and optical coherence tomography scan (E‐2) of both eyes of patient with *IMPG1* variant (c.1945C > T; p.L649F). F, Color fundus photographs (F‐1) and optical coherence tomography scan (F‐2) of both eyes of patient with *IMPG1* variant (c.1738C > T; p.R580C). G, Color fundus photographs of both eyes of patient with *IMPG1* variant (c.336TC > C; p.I112IX). H, Color fundus photographs (H‐1) and optical coherence tomography scan (H‐2) of both eyes of patient with *OTX2* variant (c.844T > A; p.C282S). I, Color fundus photographs (I‐1) and optical coherence tomography scan (I‐2) of both eyes of patient with *PRDM13* variant (c.113C > T; p.S38L). J, Color fundus photographs (J‐1) and optical coherence tomography scan (J‐2) of both eyes of patient with *PROM1* variant (c.1345G > A; p.V449M). K, Fluorescein angiogram of both eyes of patient with *PROM1* variant (c.155T > C; p.I52T), color fundus photographs were of too low quality to evaluate. L, Color fundus photographs (L‐1) and fundus autofluorescence images (L‐2) of both eyes of patient with *RP1L1* variant (c.553G > T; p.A185S)
**Figure S2.** Clinical images of patient carrying variants in autosomal recessive macular dystrophy genes previously described as pathogenic as listed in Table 2. A, Color fundus photographs (A‐1) and fundus autofluorescence images (A‐2) of both eyes of patient with *ABCA4* variant (c.6089G > A; p.R2030Q). B, Color fundus photographs (B‐1) and optical coherence tomography scan (B‐2) of both eyes of patient with *ABCA4* variant (c.3113C > T; p.A1038V). C, Color fundus photographs of both eyes of patient with *ABCA4* variant (c.2947A > G; p.T983A). D‐G, Color fundus photographs (D‐1/E/F‐1/G) and optical coherence tomography scans (D‐2 and F‐2) of both eyes of patients with *ABCA4* variant (c.2588G > C; p.G863A). H, Color fundus photographs of both eyes of patient with *ABCA4* variant (c.2546T > C; p.V849A). I, J, Color fundus photographs (I‐1 and J‐1), fundus autofluorescence images (I‐2), and fluorescein angiogram (J‐2) of both eyes of patients with *ABCC6* variant (c.2787+1G > T; p.?). K‐L, Color fundus photographs (K) of both eyes, and fluorescein angiogram (L‐1) and optical coherence tomography scan (L‐2) of the right eye of another patient with *MFSD8* variant (c.1006G > C; p.E336Q). M, Color fundus photographs (M‐1) and optical coherence tomography scan (M‐2) of both eyes of patient with *PROM1* variant (c.1355A > TA; p.Y452YX).

**Table S1.** Genes associated with age‐related macular degeneration‐mimicking diseases.

**Table S2.** Variants of unknown clinical significance or conflicting interpretations of pathogenicity.
