# Supplementary material for: Genetic screening for macular dystrophies in patients clinically diagnosed with dry age‐related macular degeneration
Source: Clin Genet. 2018 Oct 15;94(6):569–74. doi: 10.1111/cge.13447 (PMC6282796; doi:10.1111/cge.13447)
Supplement: Supplementary file 1 — Figure S1. Clinical imaging of patients with a variant of unknown clinical significance in autosomal dominant macular dystrophy genes identified in cases diagnosed with dry age‐related macular degeneration as listed in Table 1. A, Color fundus photographs (A‐1) and fluorescein angiogram (A‐2) of both eyes of patient with BEST1 variant (c.1193C > T; p.S398F). B, Color fundus photographs (B‐1) and optical coherence tomography scan (B‐2) of both eyes of patient with ELOVL4 variant (c.145A > G; p.T49A). C, Color fundus photographs (C‐1) and fluorescein angiogram (C‐2) of both eyes of patient with FSCN1 variant (c.1057G > A; p.V353M). D, Color fundus photographs (D‐1) and optical coherence tomography scan (D‐2) of both eyes of patient with IMPG1 variant (c.1982G > A; p.R661H). E, Color fundus photographs (E‐1) and optical coherence tomography scan (E‐2) of both eyes of patient with IMPG1 variant (c.1945C > T; p.L649F). F, Color fundus photographs (F‐1) and optical coherence tomography scan (F‐2) of both eyes of patient with IMPG1 variant (c.1738C > T; p.R580C). G, Color fundus photographs of both eyes of patient with IMPG1 variant (c.336TC > C; p.I112IX). H, Color fundus photographs (H‐1) and optical coherence tomography scan (H‐2) of both eyes of patient with OTX2 variant (c.844T > A; p.C282S). I, Color fundus photographs (I‐1) and optical coherence tomography scan (I‐2) of both eyes of patient with PRDM13 variant (c.113C > T; p.S38L). J, Color fundus photographs (J‐1) and optical coherence tomography scan (J‐2) of both eyes of patient with PROM1 variant (c.1345G > A; p.V449M). K, Fluorescein angiogram of both eyes of patient with PROM1 variant (c.155T > C; p.I52T), color fundus photographs were of too low quality to evaluate. L, Color fundus photographs (L‐1) and fundus autofluorescence images (L‐2) of both eyes of patient with RP1L1 variant (c.553G > T; p.A185S) Figure S2. Clinical images of patient carrying variants in autosomal recessive macular dystrophy genes prev [file CGE-94-569-s001.pdf]

Supporting information

Figure S1. **Page 2-7**

Figure S2. **Page 8-11**

Figure S1. Clinical imaging of patients with a variant of unknown clinical significance in autosomal dominant macular dystrophy genes identified in cases diagnosed with dry AMD as listed in Table 1.

A – Color fundus photographs (A-1) and fluorescein angiogram (A-2) of both eyes of patient with *BEST1* variant (c.1193C>T; p.S398F).
B – Color fundus photographs (B-1) and optical coherence tomography scan (B-2) of both eyes of patient with *ELOVL4* variant (c.145A>G; p.T49A).
C – Color fundus photographs (C-1) and fluorescein angiogram (C-2) of both eyes of patient with *FSCN1* variant (c.1057G>A; p.V353M).
D – Color fundus photographs (D-1) and optical coherence tomography scan (D-2) of both eyes of patient with *IMPG1* variant (c.1982G>A; p.R661H).
E – Color fundus photographs (E-1) and optical coherence tomography scan (E-2) of both eyes of patient with *IMPG1* variant (c.1945C>T; p.L649F).
F – Color fundus photographs (F-1) and optical coherence tomography scan (F-2) of both eyes of patient with *IMPG1* variant (c.1738C>T; p.R580C).
G – Color fundus photographs of both eyes of patient with *IMPG1* variant (c.336TC>C; p.I112IX).
H – Color fundus photographs (H-1) and optical coherence tomography scan (H-2) of both eyes of patient with *OTX2* variant (c.844T>A; p.C282S).
I – Color fundus photographs (I-1) and optical coherence tomography scan (I-2) of both eyes of patient with *PRDM13* variant (c.113C>T; p.S38L).
J – Color fundus photographs (J-1) and optical coherence tomography scan (J-2) of both eyes of patient with *PROM1* variant (c.1345G>A; p.V449M).
K – Fluorescein angiogram of both eyes of patient with *PROM1* variant (c.155T>C; p.I52T), color fundus photographs were of too low quality to evaluate.
L – Color fundus photographs (L-1) and fundus autofluorescence images (L-2) of both eyes of patient with *RP1L1* variant (c.553G>T; p.A185S).

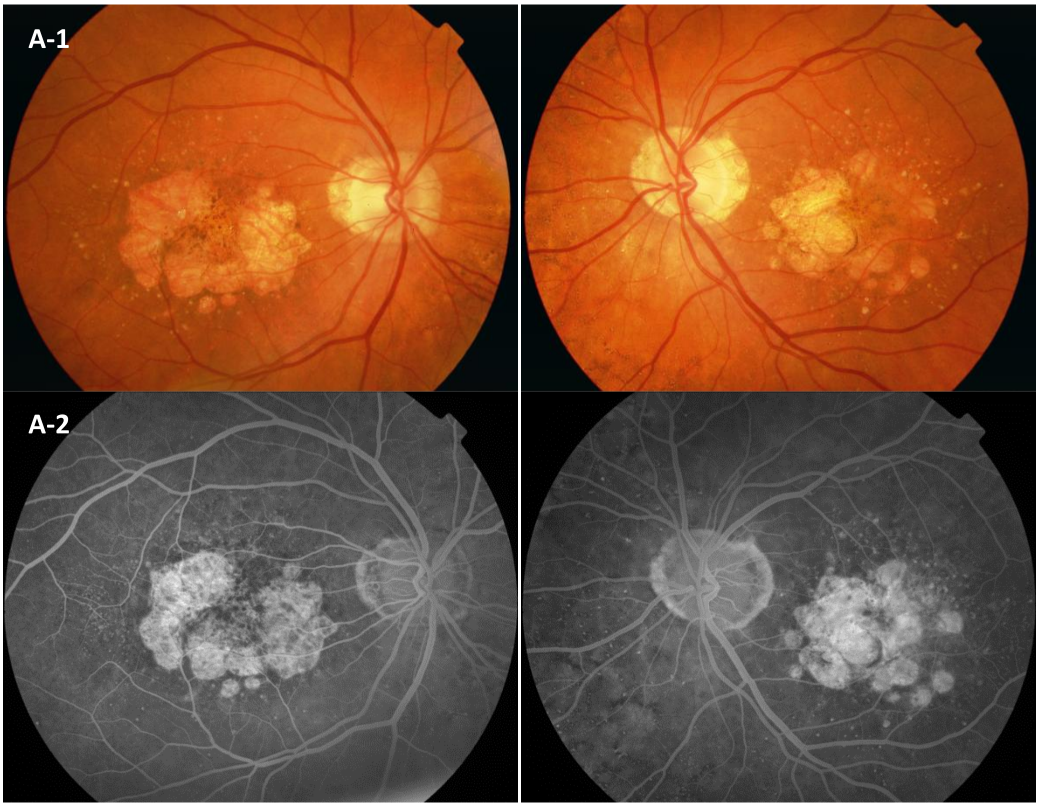

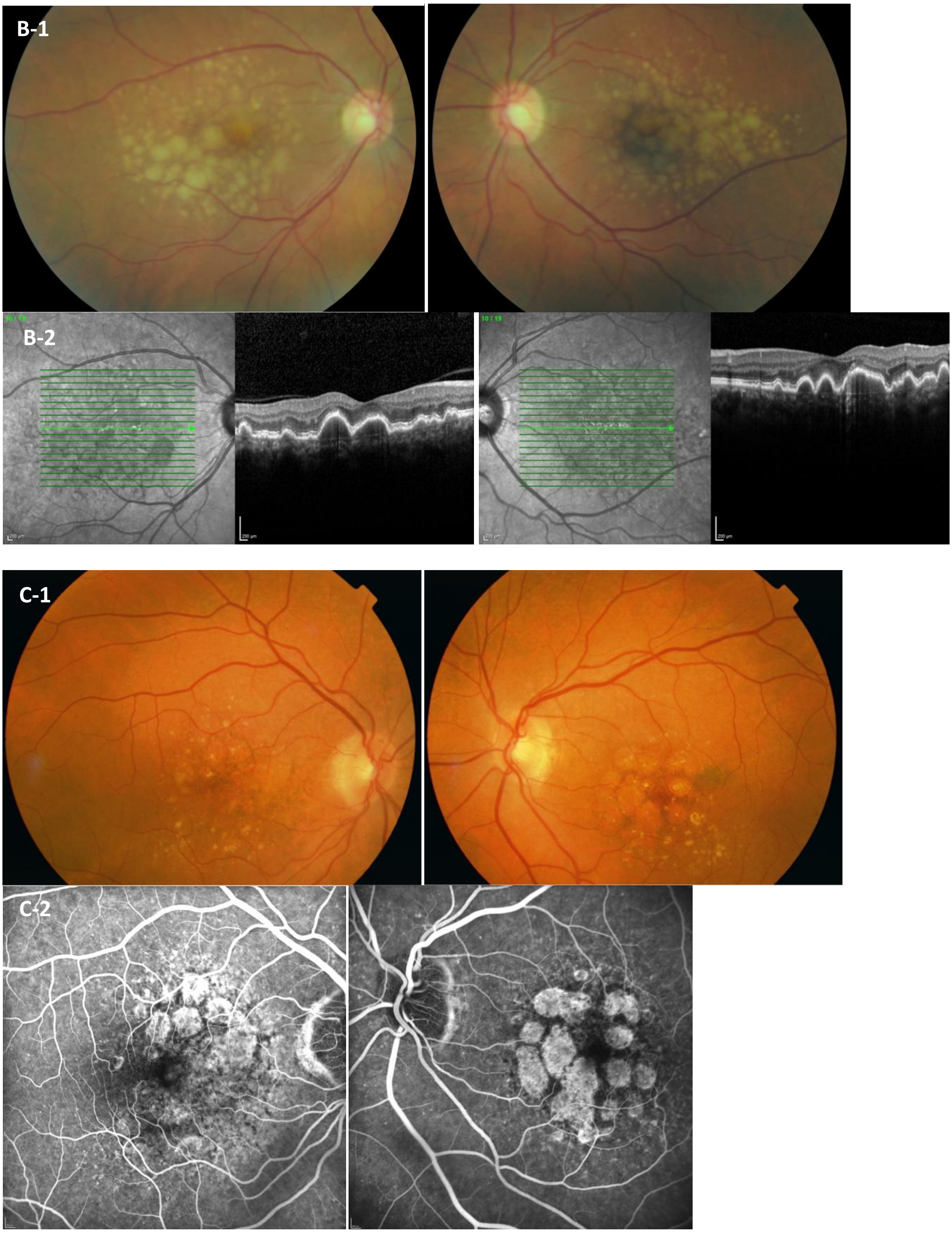
B-1
B-2
C-1
C-2

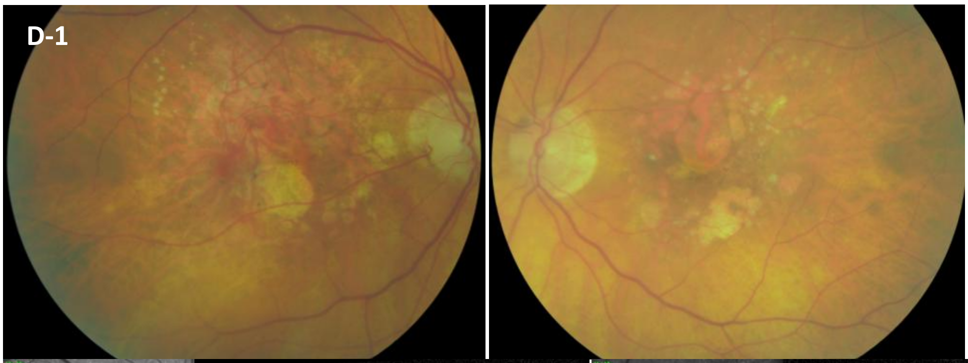
D-1

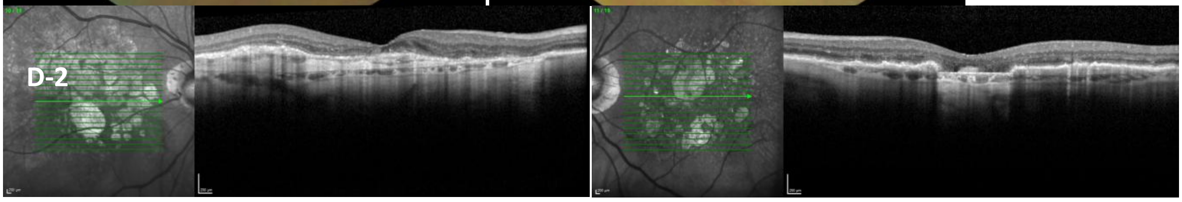
D-2

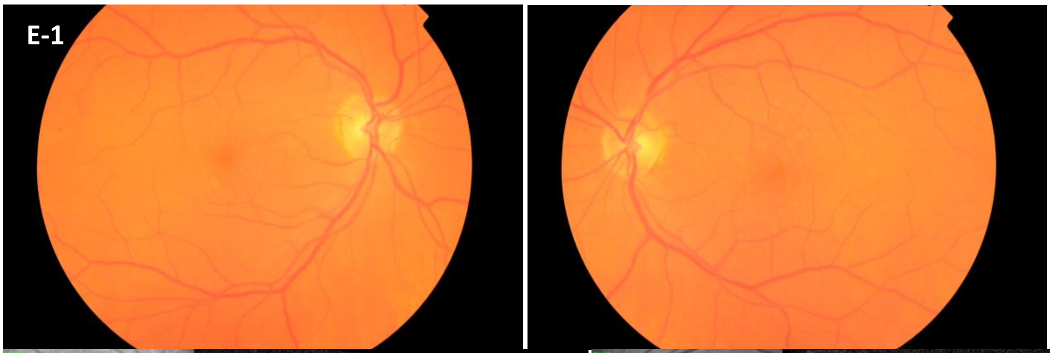
E-1

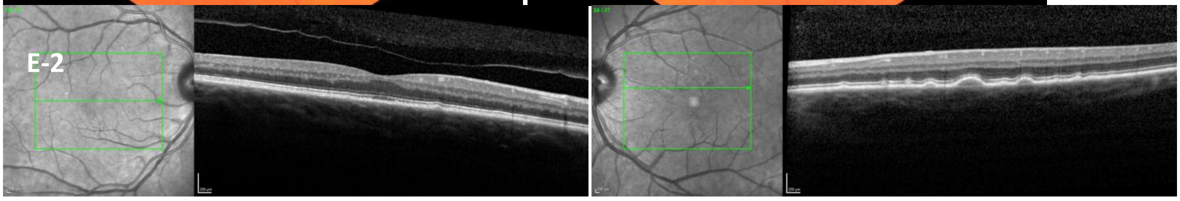
E-2

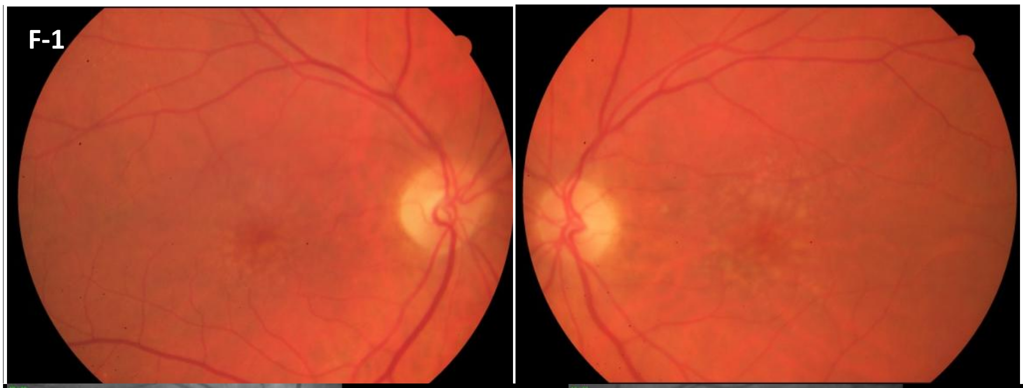
F-1

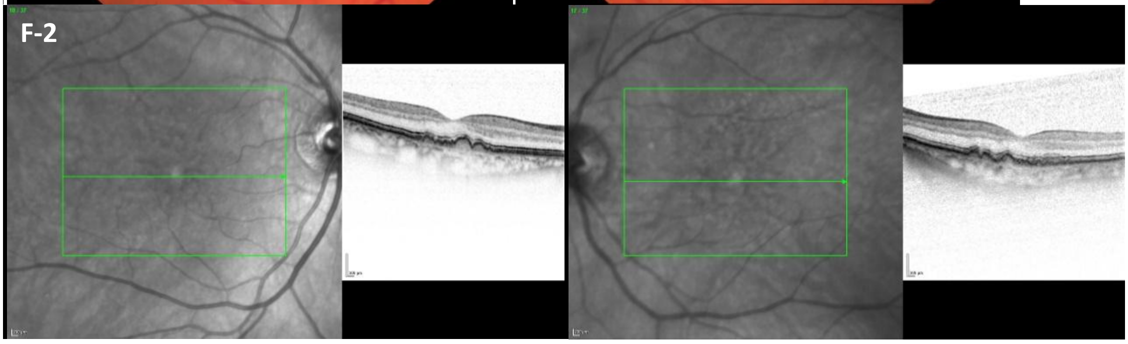
F-2

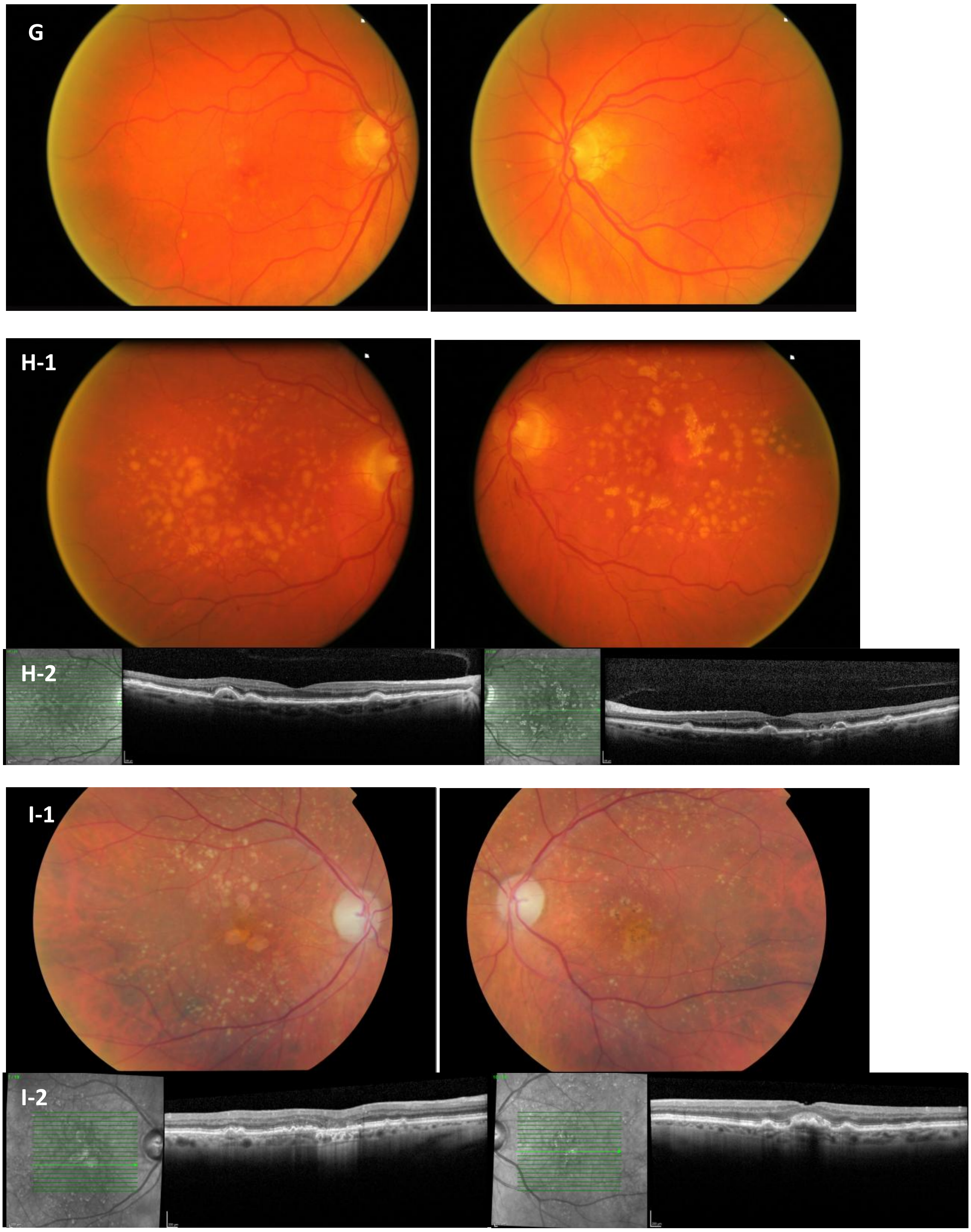
G
H-1
H-2
I-1
I-2

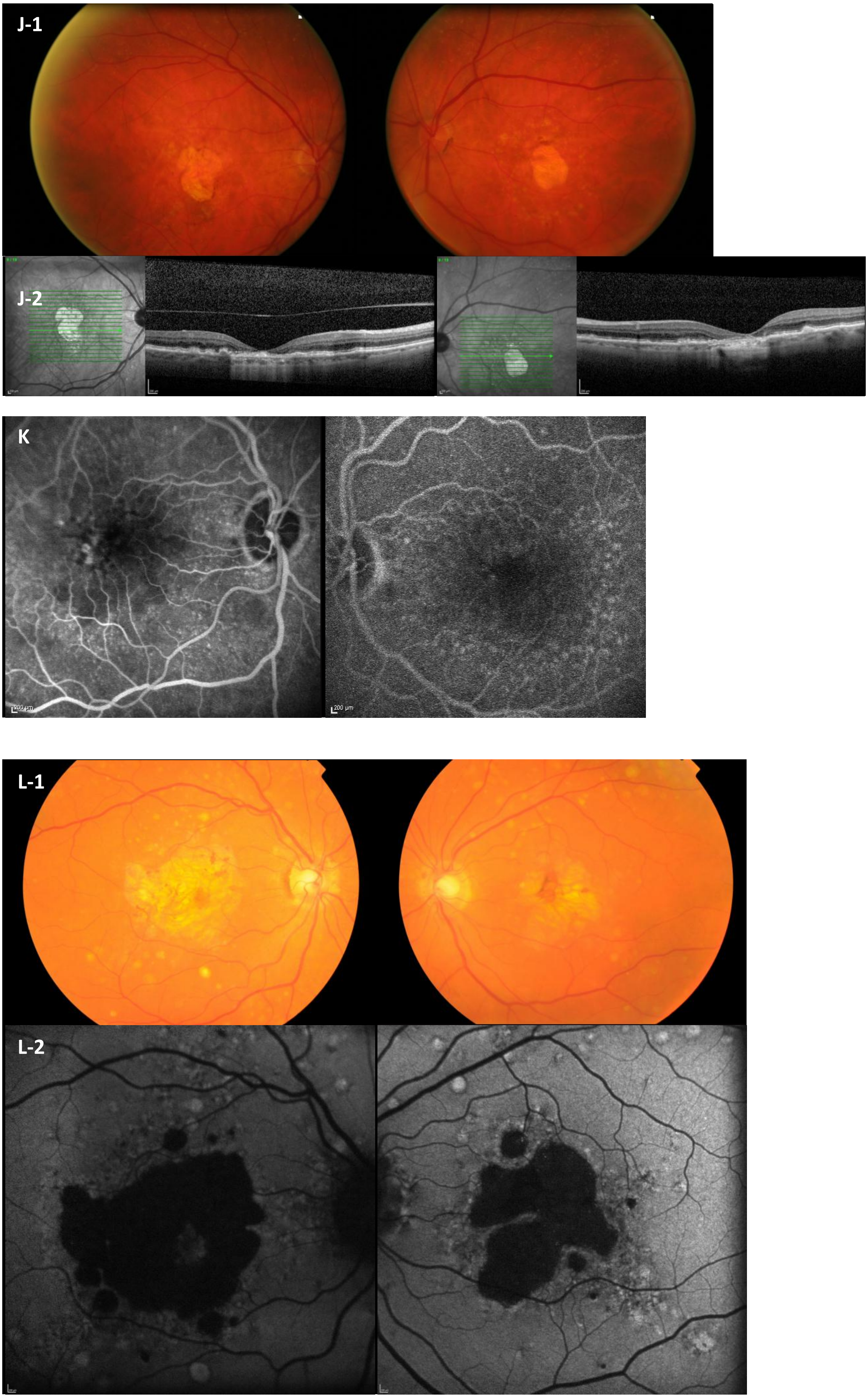
J-1
J-2
K
200 µm
200 µm
L-1
L-2

Figure S2. Clinical images of patient carrying variants in autosomal recessive macular dystrophy genes previously described as pathogenic as listed in Table 2.

A – Color fundus photographs (A-1) and fundus autofluorescence images (A-2) of both eyes of patient with *ABCA4* variant (c.6089G>A; p.R2030Q).
B – Color fundus photographs (B-1) and optical coherence tomography scan (B-2) of both eyes of patient with *ABCA4* variant (c.3113C>T; p.A1038V).
C – Color fundus photographs of both eyes of patient with *ABCA4* variant (c.2947A>G; p.T983A).
D – G – Color fundus photographs (D-1/E/F-1/G) and optical coherence tomography scans (D-2 and F-2) of both eyes of patients with *ABCA4* variant (c.2588G>C; p.G863A).
H – Color fundus photographs of both eyes of patient with *ABCA4* variant (c.2546T>C; p.V849A).
I – J – Color fundus photographs (I-1 and J-1), fundus autofluorescence images (I-2), and fluorescein angiogram (J-2) of both eyes of patients with *ABCC6* variant (c.2787+1G>T; p.?).
K – L – Color fundus photographs (K) of both eyes, and fluorescein angiogram (L-1) and optical coherence tomography scan (L-2) of the right eye of another patient with *MFSD8* variant (c.1006G>C; p.E336Q).
M – Color fundus photographs (M-1) and optical coherence tomography scan (M-2) of both eyes of patient with *PROM1* variant (c.1355A>TA; p.Y452YX).

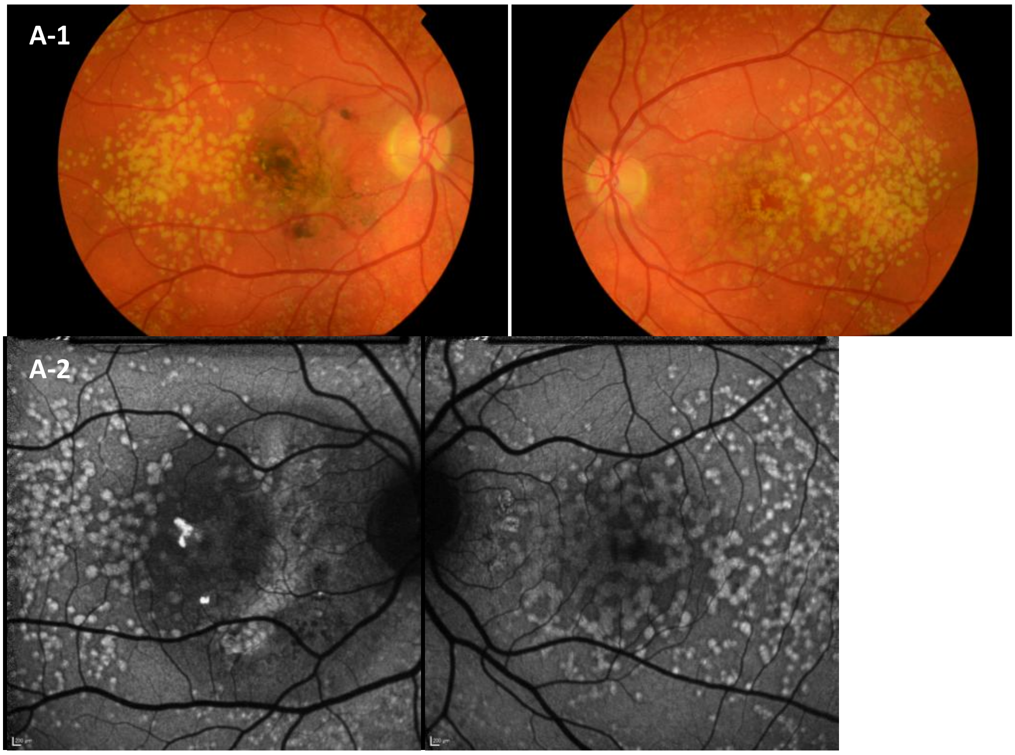

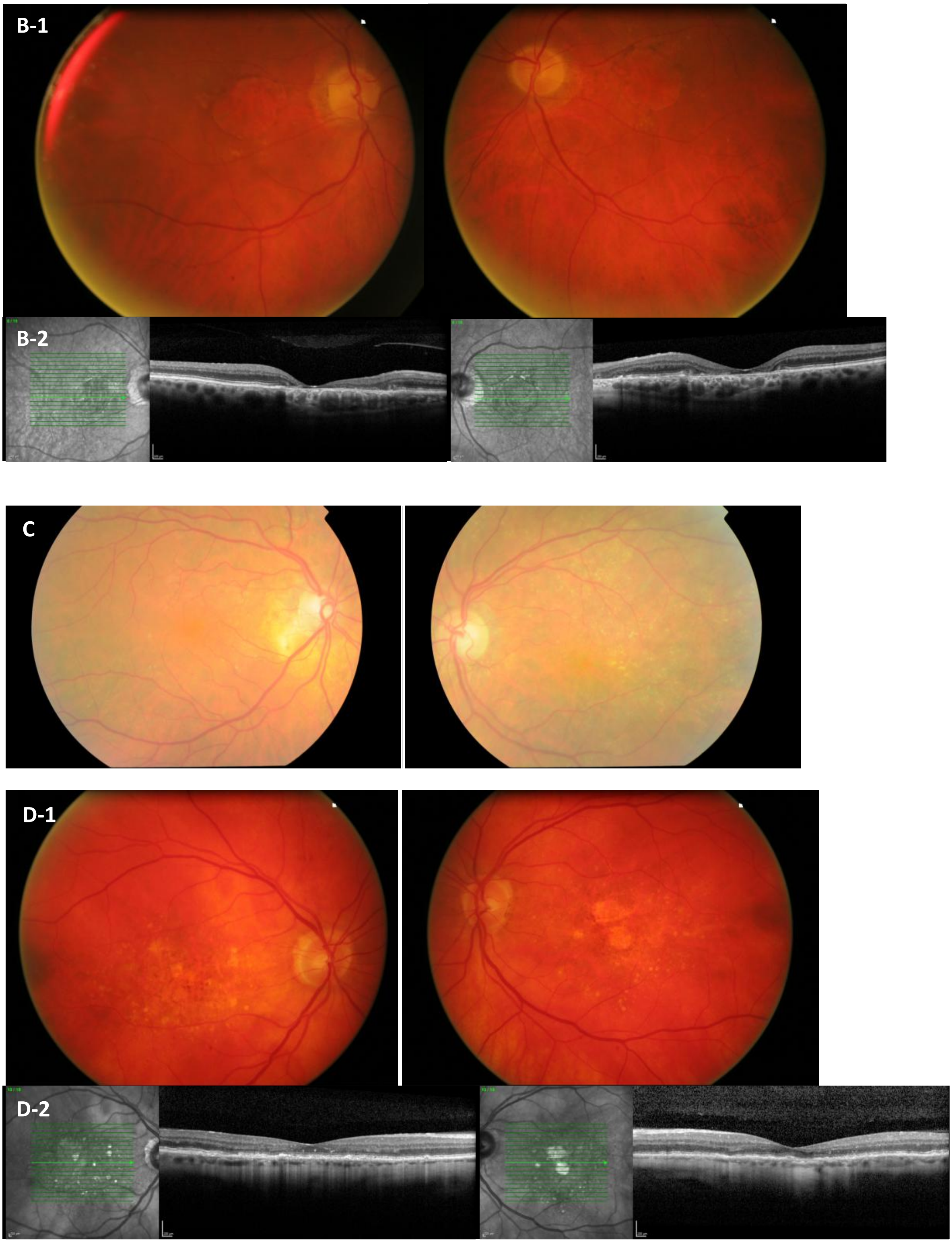
B-1
B-2
C
D-1
D-2

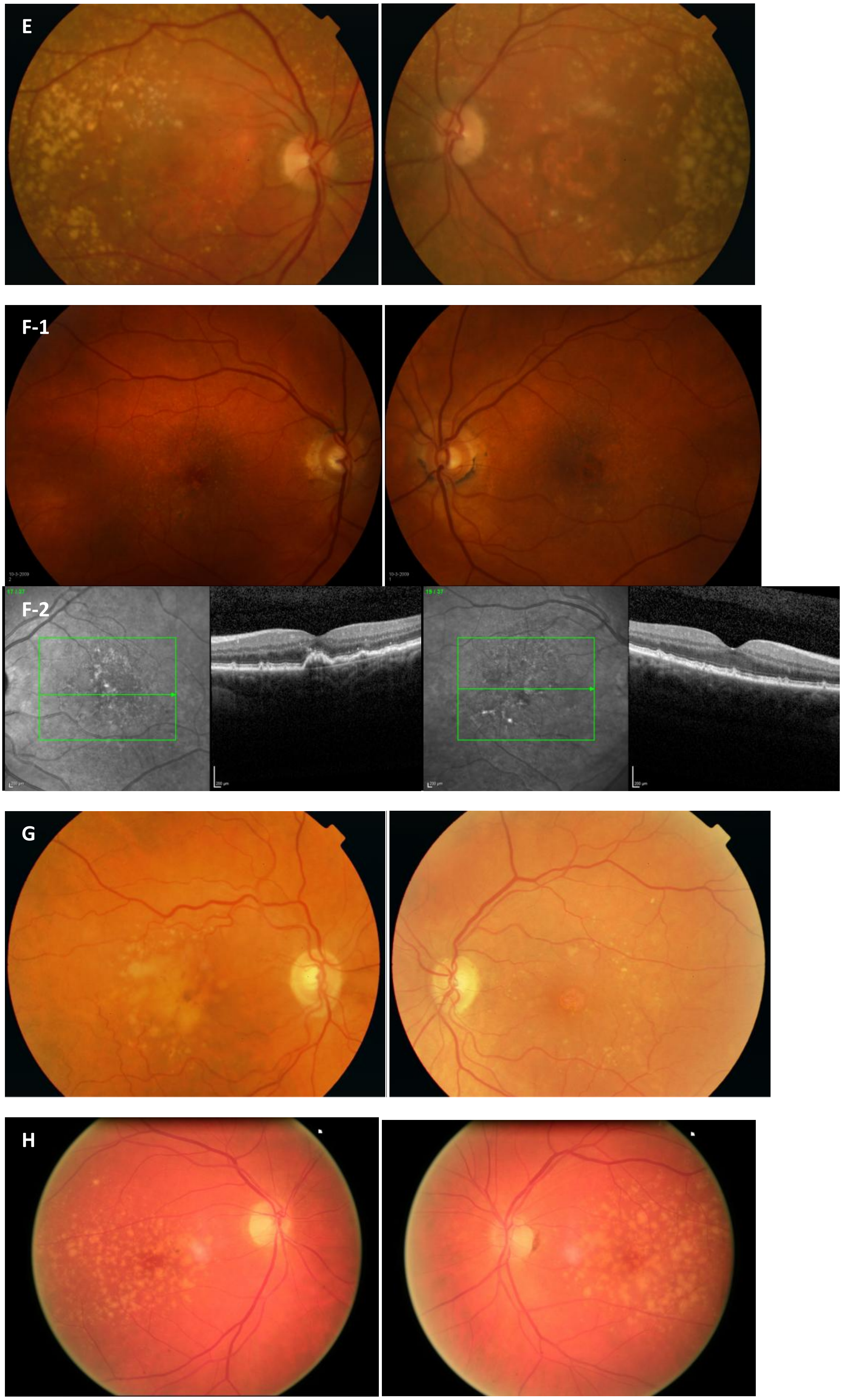
E
F-1
F-2
G
H

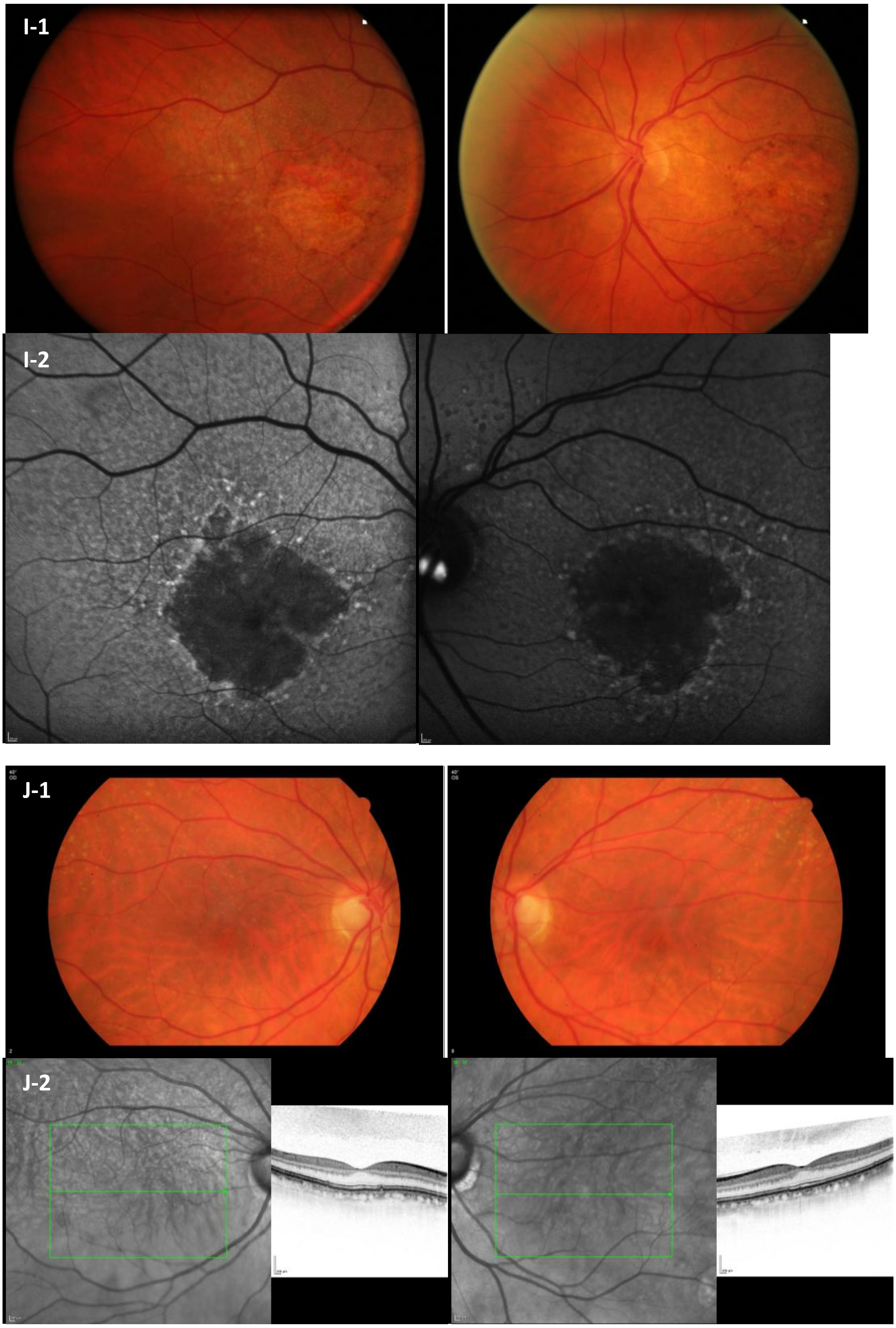
I-1
I-2
J-1
J-2

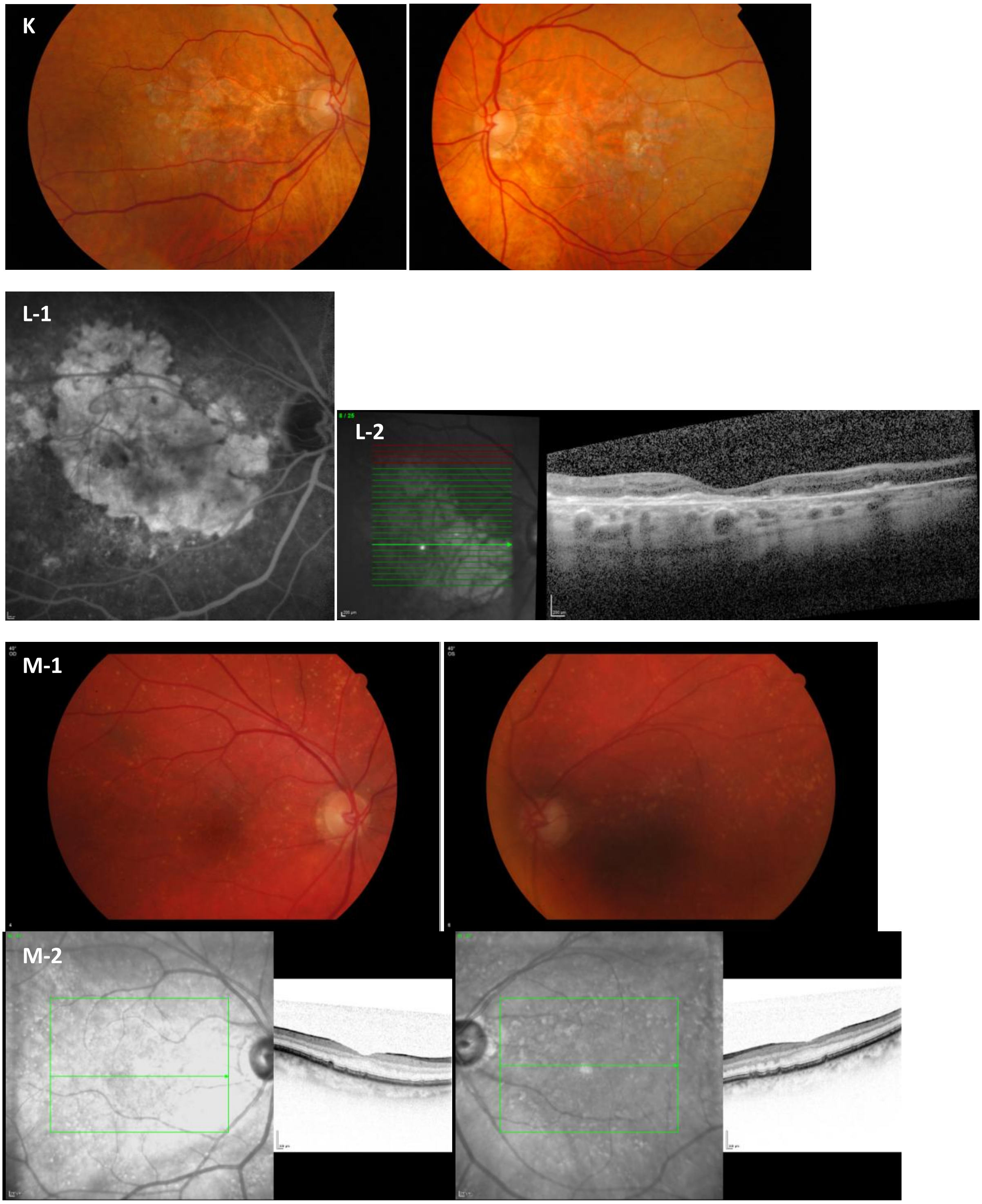
K
L-1
L-2
M-1
M-2
