# Supplementary material for: Genetic screening for macular dystrophies in patients clinically diagnosed with dry age‐related macular degeneration
Source: Clin Genet. 2018 Oct 15;94(6):569–74. doi: 10.1111/cge.13447 (PMC6282796; doi:10.1111/cge.13447)
Supplement: Supplementary file 2 — Table S1. Genes associated with age‐related macular degeneration‐mimicking diseases. [file CGE-94-569-s002.docx]

**Supplementary table 1. Genes associated with AMD-mimicking diseases.**

| **Gene** | **Chr.** | **Associated disease** |
| --- | --- | --- |
| **Autosomal Dominant** | | |
| *BEST1* | 11 | Adult-onset foveomacular vitelliform dystrophy (AFVD)  Best vitelliform macular dystrophy (BVMD) |
| *C1QTNF5/MFRP* | 11 | Late onset retinal degeneration (LORD) |
| *CTNNA1* | 5 | Butterfly-shaped pigment dystrophy |
| *EFEMP1* | 2 | Malattia Leventinese (ML)/Doyne honeycomb retinal dystrophy |
| *ELOVL4* | 6 | Stargardt-like macular dystrophy (STGD3)  Autosomal dominant macular dystrophy |
| *FSCN2* | 17 | Autosomal dominant macular degeneration  Autosomal dominant retinitis pigmentosa |
| *GUCA1B* | 6 | Autosomal dominant retinal degeneration |
| *OTX2* | 14 | Autosomal dominant pattern dystrophy |
| *PRDM13* | 6 | North-Carolina macular dystrophy (NCMD) |
| *PRPH2* | 6 | Central areolar choroidal dystrophy  Adult-onset foveomacular vitelliform dystrophy  Autosomal dominant pattern dystrophy  Pseudo-Stargardt pattern dystrophy |
| *RP1L1* | 8 | Autosomal dominant occult macular dystrophy |
| *TIMP3* | 22 | Sorsby fundus dystrophy |
| **Autosomal Recessive** | | |
| *ABCA4* | 1 | (late-onset) Stargardt disease |
| *ABCC6* | 16 | Pseudoxanthoma elasticum related dystrophy (angioid streaks) |
| *DRAM2* | 1 | Autosomal recessive macular dystrophy |
| *MFSD8* | 4 | Nonsyndromic autosomal recessive macular dystrophy |
| **Autosomal Dominant or Autosomal Recessive** | | |
| *IMPG1* | 6 | Autosomal dominant benign concentric annular macular dystrophy  Autosomal dominant and autosomal recessive vitelliform macular dystrophies |
| *PROM1* | 4 | Autosomal dominant bull's-eye macular dystrophy  Autosomal dominant stargardt-like dystrophy  Autosomal recessive cone-rod dystrophy |

Based on Saksens et al. 2014 (Prog Retin Eye Res 39:23-57) and RetNet, the Retinal Information Network.
